# Effect of thermal cycling on the flexural strength of 3-D printed, CAD/CAM milled and heat-polymerized denture base materials

**DOI:** 10.1186/s12903-024-04122-y

**Published:** 2024-03-20

**Authors:** Tuğba Temizci, Hatice Nalan Bozoğulları

**Affiliations:** Department of Prosthodontics, Faculty of Dentistry, Karamanoğlu Mehmetbey University, Karaman, Turkey

**Keywords:** Denture base, 3D printed, Milled, Flexural strength

## Abstract

**Background:**

This study compared the impact of thermal cycling on the flexural strength of denture-base materials produced through conventional and digital methods, using both subtractive and additive approaches.

**Methods:**

In total, 60 rectangular specimens were fabricated with specific dimensions for flexural strength tests. The dimensions were set according to the International Organization for Standardization (ISO) guideline 20795-1:2013 as 64 × 10 × 3.3 ± 0.2 mm. Specimens from each material group were divided into two subgroups (thermal cycled or nonthermal cycled, *n* = 10/group). We used distinct methods to produce three different denture-base materials: Ivobase (IB), which is a computer-aided-design/computer-aided-manufacturing-type milled pre-polymerized polymethyl methacrylate resin disc; Formlabs (FL), a 3D-printed denture-base resin; and Meliodent (MD), a conventional heat-polymerized acrylic. Flexural strength tests were performed on half of the samples without a thermal-cycle procedure, and the other half were tested after a thermal cycle. The data were analyzed using a two-way analysis of variance and a post hoc Tukey test (α = 0.05).

**Results:**

Based on the results of flexural-strength testing, the ranking was as follows: FL > IB > MD. The effect of thermal aging was statistically significant for the FL and IB bases, but not for the MD base.

**Conclusions:**

Digitally produced denture bases exhibited superior flexural strength compared with conventionally manufactured bases. Although thermal cycling reduced flexural strength in all groups, the decrease was not statistically significant in the heat-polymerized acrylic group.

## Background

Removable partial and complete dentures play a significant role in restoring aesthetic and functional characteristics after total and partial tooth loss. Polymethyl methacrylate (PMMA) is among the most commonly used materials for denture bases. Heat-polymerized polymethyl methacrylate, commonly referred to as acrylic resin, is favored due to its availability, aesthetic qualities, biocompatibility, cost-effectiveness, lightweight nature, relative ease of repair, and simple processability [1–4]. However, the durability of acrylic-resin-based denture bases is influenced by several factors, such as the powder-liquid ratio, polymerization method (rapid boil or slow boil), knowledge and skill of the technician, and material storage conditions. Nevertheless, challenges persist in using heat-polymerized acrylic dentures. Thermal shrinkage due to the polymerization process can lead to inadequate adaptation of the denture base to surrounding tissues [5]. Other issues include allergic reactions caused by residual monomers [4], low durability, inadequate surface hardness, and poor wear resistance, prompting an ongoing search for an ideal denture-base material [1, 2].

With computer-aided-design (CAD) and computer-aided-manufacturing (CAM) systems, it is possible to manufacture complete dentures from fully polymerized or pre-polymerized acrylic discs using subtractive methods, such as milling, or additive methods, including 3D printing with acrylic resins. CAD-CAM technology can enable production of high-precision dental prosthetics more quickly and with less discomfort to the patient compared to traditional methods. Additionally, it allows direct duplication of an existing denture [6, 7].

Milling is more common in denture-base production than 3D printing [6]. The accuracy of dentures produced through milling depends on the materials used and the milling tools (number and size of milling cutters) employed [8, 9]. The denture-base material obtained through milling is highly durable compared to other options because it has fewer structural flaws and contains inorganic fillers in the final stage [7, 9]. However, fabrication of dentures using milling generates significant amounts of waste and requires large quantities of raw materials [10].

3D printing is achieved through application of a material in consecutive layers. It is considered less costly than milling because of reduced material waste and absence of tool wear [11]. 3D printing also allows simultaneous production of multiple objects and printing of intricate and complex designs [12, 13]. Although complete dentures produced using 3D printers offer a viable treatment alternative for total tooth loss, their adoption in clinics is not yet widespread due to the relatively high costs of the equipment and materials, limited accessibility to the materials, and a lack of extensive research on their mechanical and physical properties.

Flexural strength is measured based on the highest bending stress experienced by a material at the point of fracture. Dentures are subjected to flexural stresses during chewing, which can lead to deformation or breakage over long periods [14]. Therefore, high flexural strength is necessary to prevent sudden failure [15]. As per the American Dental Association Standard No. 139, and in accordance with the International Organization for Standardization (ISO) guidelines 20795-1 for denture-base polymers, a flexural strength test is commonly used to assess material resistance [16–18].

The oral environment is a thermally dynamic setting. Considering that temperature fluctuations can impact the properties of a material, it is essential to test the mechanical behavior of denture-base materials under conditions that mimic intraoral conditions. Thermal cycling is the preferred method of testing responses to such conditions.

A literature search revealed few publications that reported on the mechanical properties of the photopolymerized acrylic resin esters used in 3D printing, and only one study that investigated the effects of thermal cycling [1]. Thus, there is a need for more studies investigating the potential of 3D denture-base materials and manufacturing techniques for complete dentures. Therefore, in this study, we compared the impact of thermal cycling on the flexural strength of denture-base materials produced through conventional and digital methods, using both subtractive and additive approaches. Our primary hypothesis was that there would be differences among the materials, and our secondary hypothesis was that thermal cycling would influence the flexural strength of the materials.

## Materials and methods

In total, 60 rectangular specimens were fabricated for flexural-strength tests with specific dimensions set according to the ISO 20795-1:2013 standards. Specifically, the specimens were 64 × 10 × 3.3 ± 0.2 mm. Specimens from each material group were divided into two subgroups (thermal cycled or nonthermal cycled, *n* = 10/group). The properties of the materials and manufacturers are presented in Table 1.

**Table 1:** Materials used in the present study

Material	Abbr	Manufacturer	Denture base fabrication technique	Composition
IvoBase CAD	IB	Ivoclar Vivadent, Schaan, Liechtenstein	CAD/CAM milling	Prepolymerized PMMA discs 50–100% methyl methacrylate 2.5–10% 1,4-butanediol dimethacrylate
Formlabs	FL	Somerville, MA, USA	3D Printing	55–75% w/w urethane dimethacrylate, 15–25% w/w methacrylate monomers, and < 0.9% w/w phenyl bis(2,4,6- trimethylbenzoyl)-phosphine oxide
Meliodent	MD	Kulzer, Berkshire, Germany	Conventional; heat-polymerized	Powder: Methyl methacrylate, Ethyl hexyl acrylate, N-octyl methacrylate Liquid: Methyl methacrylate, glycol dimethacrylate, dimethyl p-toluidine

We used the Fusion 360 CAD software program (Autodesk, Mill Valley, CA, USA) to design a rectangular 3D model (64 mm × 10 mm x 3.3 mm) for the CAD-CAM 3D-printed specimens (termed IB specimens). This digital design was exported to produce the specimens using a Standard Tessellation Language (STL) file. The CAD-CAM specimens were produced from a pre-polymerized PMMA resin disc using a five–axis milling machine (HinriMill 5, Goslar, Germany) and an IvoBase CAD system (Ivoclar).

The 3D-printed specimens (termed FL specimens) were produced using stereolithography (SLA) 3D printing technology (Form 3B+, FormLabs, Somerville, MA, USA) and commercially available 3D-printed denture-base resin (Formlabs denture-base resin). The layer thickness of each specimen was set at 50 μm and the build orientation was 90 degrees. The printed specimens were washed with 90% isopropyl alcohol for 3 min using an ultrasonic cleansing system (Form Wash, Formlabs, Somerville, MA, USA) and then subjected to a post-polymerization process using FormCure (Formlabs Inc., Somerville, MA, USA) for 30 + 30 min at 60 °C.

**Figure 1.**
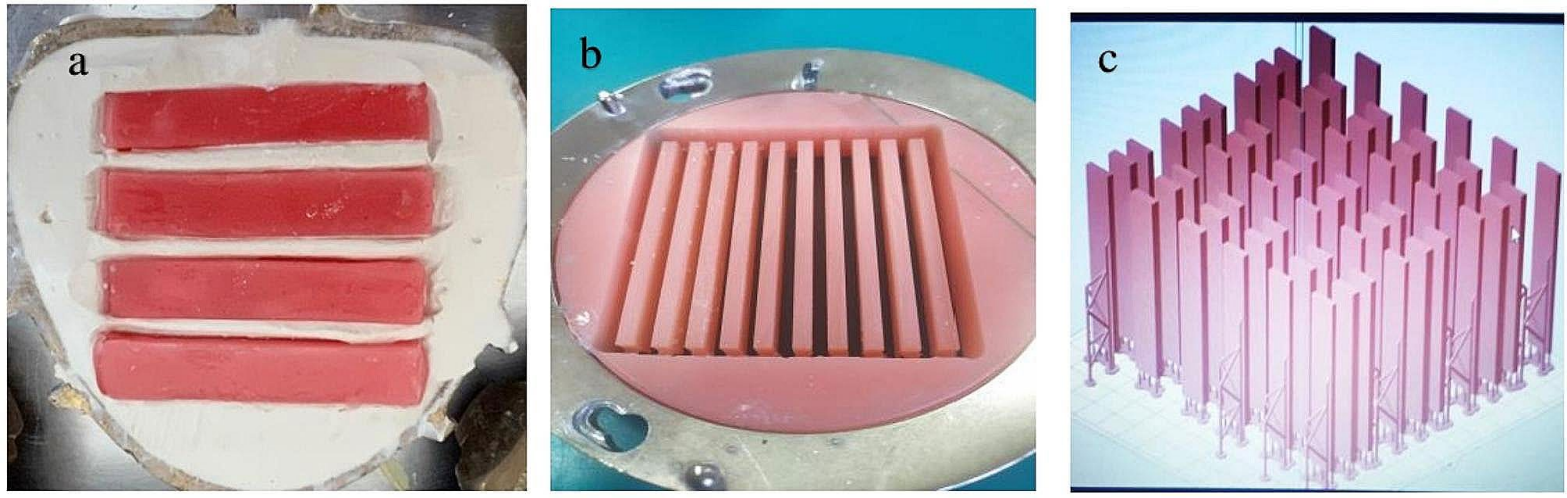
**a**. Preparation of MD samples **b**. Preparation of IB samples **c**. Preparation of FL samples

To generate specimens using the conventional method (termed MD specimens), prepared wax samples were placed in a flask. After complete hardening of the gypsum, the flasks were placed in boiling water to remove the wax. After removal of the wax, the negative voids were isolated with lacquer, and a heat-polymerized acrylic resin material (Meliodent Heat Cure) was applied according to the manufacturer’s instructions. The flasks were then subjected to 100 bars of pressure to eliminate excess acrylic material and left under 200 bars of pressure for 5 min. Once the flasks had been secured, they were placed in cold water and allowed to boil. When the temperature reached 100 °C, they were left to boil for 20 min. The samples were then removed from the flasks and leveled using a precision grinder and a hard mill. To mimic the denture surfaces, the samples were wet-ground at a speed of 60 rpm using a grinding and polishing device (Grıpo 2 V, METKON, Grinder-Polisher) with 400–600–800 grit sandpaper, consecutively. To ensure standardization of the samples during sanding and polishing, measurements were made using an electronic caliper in a systematic manner. Following sanding, polishing was carried out with polishing paste (Ivoclar Vivadent Universal Polishing Paste) and felt (Fig. 1).

### Thermal cycling procedure

The specimens exposed to thermal cycling underwent a thermocycling procedure consisting of 5000 cycles in a distilled-water bath at temperatures of 5–55 °C. Each cycle lasted 60 s and involved the following steps: 20 s in a 5 °C bath, a 10-second transfer of the samples to another bath, 20 s in a 55 °C bath, and a 10-second transfer back to the 5 °C bath.

### Flexural strength test

**Figure 2.**
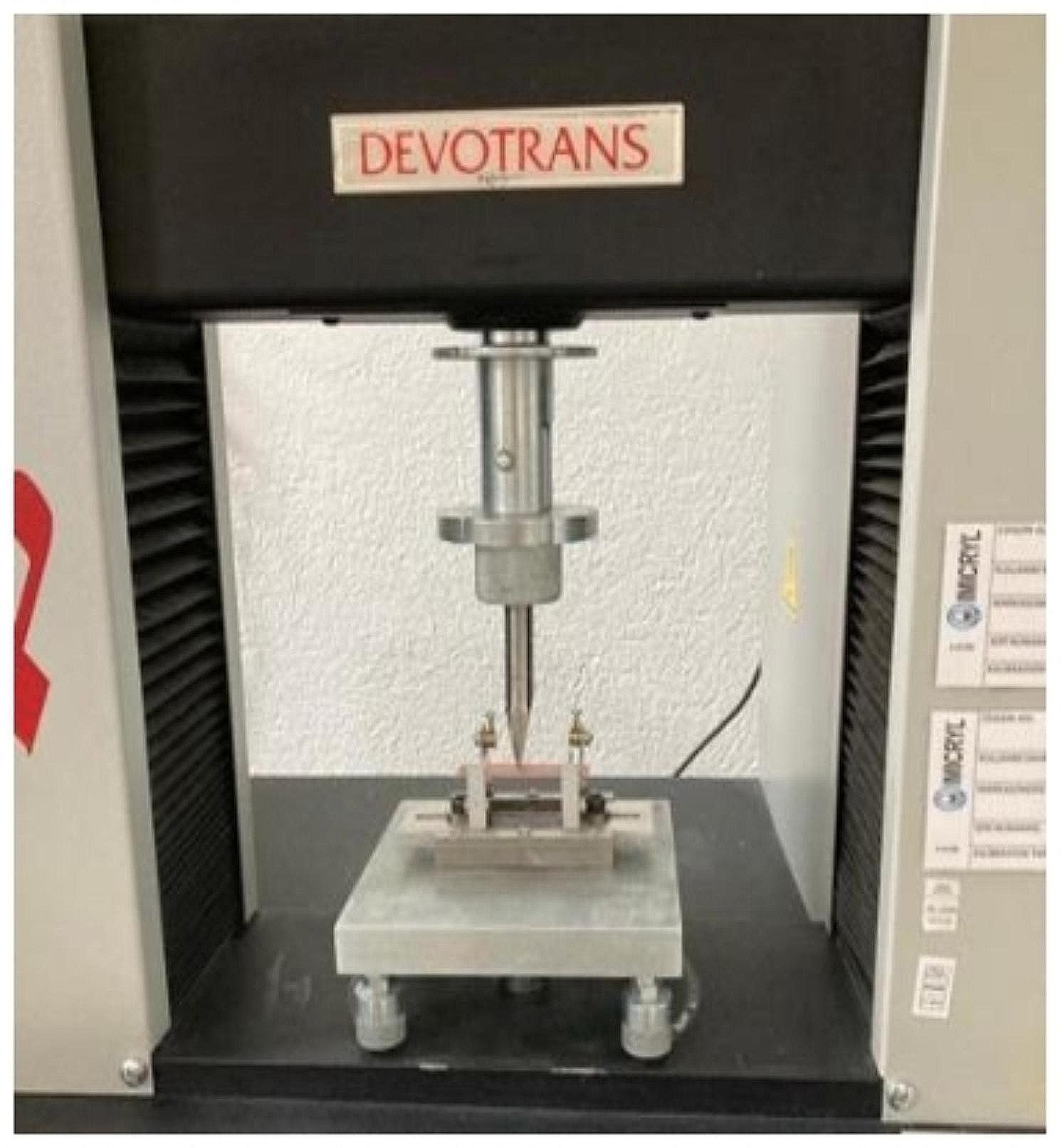
Acrylic specimen loaded on universal testing machine

The flexural strength was tested using a three-point flexure test on a universal testing machine. The specimens were placed symmetrically on the base of the testing machine (Devotrans, Türkiye) (Fig. 2). The load force started at 0 and increased evenly via a steady shift of 5 ± 1 mm/min until the specimen cracked. The flexural strength of each specimen was measured according to the following formula:

FS = 3FL/2bh^2^.

where FS is the flexural strength (MPa), F is the maximum force applied to the specimen (N), L is the distance between the specimen carriers (mm), b is the specimen width (mm), and h is the specimen height (mm).

The data were analyzed using a two-way analysis of variance (ANOVA, IBM SPSS 20.0 software; SPSS Inc., Chicago, IL) and a Tukey honest post hoc test was applied to detect differences among the groups. The statistical significance level was set at *p* < 0.05.

## Results

When the results of the flexural strength test were examined, we found that the resin materials produced via CAD/CAM performed better than those in the traditional heat-polymerized acrylic group. Before thermal cycling, the highest flexural strength values were in the FL group (113.53 ± 7.94 MPa), followed by the IB group (104.65 ± 5.12 MPa) and the MD group (232.67 ± 32.60 MPa). Thermal cycling decreased flexural strength in all groups. The effect of thermal aging was statistically significant for the FL and IB groups, but not the MD group (Table 2).

**Table 2:** Descriptive statistics (mean ± standard deviations) of flexural strength (MPa) values

Material	Non-aging	Aging
IvoBase CAD	104.65 ± 5.12^b^	95.95 ± 5.64^cd^
Meliodent	94.23 ± 10.40^d^	91.39 ± 9.92^d^
Formlabs	113.53 ± 7.94^a^	103.05 ± 12.84^bc^

Different superscript letters indicate significant differences (*p* < 0.05)

According to the results of the two-way ANOVA, material type and aging were not statistically significant. However, when the material type and aging were evaluated together, this comparison became statistically significant (*p* < 0.05) (Table 3).

**Table 3:** Results of two-way anova for flexural strength

Test method	Source of variation	Sum of squares	df	Mean square	F	*p*
	Material	2357.233	2	1178.617	4.499	0.182
	Aging	437.400	1	437.400	1.670	0.325
	Material*Aging	523.900	2	261.950	3.249	0.047
	Error	4354.400	54	80.637		
	Total	606417.067	60			

*p* < 0,05

## Discussion

This in vitro study compared the flexural strength of denture-base materials produced by different methods by investigating the effect of thermal cycling. The flexural strength of the materials differed among the three groups, supporting our first hypothesis. However, thermal cycling did not result in a statistically significant change in any group, so the second hypothesis was rejected.

For successful prosthetic treatment and patient satisfaction, denture-base materials need to possess sufficient flexural strength [1]. Dentures with acrylic-resin bases are subject to a range of impact forces, including dropping the device when outside the mouth or repeated chewing forces when inside the mouth, and are prone to breakage caused by bending fatigue [19]. The denture base, which is subjected to bending stress during chewing, is supported by alveolar crests with uneven bone resorption [1, 20].

A previous study compared a three-point bending test with a four-point bending test for various polymers [21]. The authors reported that the three-point bending test produced statistically higher and more reliable bending-strength values than the four-point bending test.

ISO guideline 20795-1 specifies the three-point bending test as the standard for measuring the mechanical properties and clinical performance of polymer-based materials [20, 22]. Therefore, we measured the flexural strength of the materials using the three-point bending test. This guideline indicates that acrylic resins should achieve a minimum flexural strength of 65 MPa. Based on this criterion, all groups in our study were suitable for clinical use.

Acrylic denture bases produced using conventional methods have long been the industry standard. Accordingly, numerous studies have been conducted on heat-polymerized acrylics. In this study, the flexural strength of conventionally manufactured bases was 94.23 MPa, which is consistent with previous findings. However, previous comparisons of flexural strength between CAD-CAM and conventionally produced bases yielded differing results [23–25].

Pre-polymerized PMMA-based acrylic resin disks are used during subtractive manufacturing. Because of the high temperature and pressure values during polymerization, these disks have a dense structure with low-porosity and strong cross-linking, distinguishing them from conventionally produced PMMA bases [26, 27]. Bases produced via traditional processing techniques exhibit linear shrinkage (0.45–0.9%), while digital processing eliminates excessive shrinkage [23, 27]. Numerous studies have shown that, compared with traditional acrylic-based dentures, those produced using the CAD-CAM subtractive method exhibit superior mechanical durability and surface characteristics [24, 28], have lower residual monomer content [29], and offer better tissue compatibility [30]. This is consistent with our finding that the bases in the milled group demonstrated superior flexural strength compared with those in the conventional heat-polymerized acrylic group.

Denture production via milling is more popular than additive manufacturing (i.e., 3D printing). However, 3D printing is more cost-effective due to its additive nature. Furthermore, the subtractive method is associated with wear on rotating tools, material waste, and the inability to produce multiple products simultaneously [31, 32]. 3D printing involves the application of a fluid resin material, layer by layer, onto a support structure, followed by polymerization of each layer using visible light, ultraviolet light, heat, or a laser [33]. In this process, resin layers are added sequentially until the proposed dental prosthesis form is completed. In dentistry, the use of CAD-CAM additive manufacturing is increasing for the production of fixed prosthetics, surgical guides, occlusal appliances, and complete dentures [33, 34]. In the literature, studies on acrylic resins produced through 3D printing have obtained varying results. Prpic et al. [35] measured the flexural strength of three different CAD-CAM discs, three different heat-polymerized acrylic resins, 3D printing resins, and polyamide materials using a three-point bending test. They concluded that the 3D printing resin had the lowest flexural strength compared with all other materials. Gad et al. [1] compared the flexural strength of heat-polymerized denture-base resin and 3D printing resin before and after thermal cycling. They found that the 3D printing resin exhibited lower flexural strength compared with all groups of heat-polymerized resin, both before and after thermal cycling. In our study, the specimens in the 3D printing group demonstrated the highest flexural strength. Successful use of 3D printing for denture production depends on various factors. Differences in the chemical composition of the resins used in other studies, variations among 3D printers, and different post-curing times could explain the varying results among studies. It should also be noted that the degree of polymerization is dependent on multiple factors such as the sample composition, photoinitiator concentration, sample geometry, and polymerization environment [23].

One study examined the effects of different post-curing times and parameters on the physicochemical, mechanical, and biological compatibility of 3D-printed denture bases [36]. During printing, liquid resin contains monomers and photoinitiators that are activated by UV light. The light converts the monomers into polymers, forming bonded chains at a macromolecular level [37]. However, the rapid layer-by-layer building can lead to insufficient curing density in each added layer, ultimately minimizing the efficiency of extended chain cross-linking. Therefore, a final curing cycle is applied to convert partially cured monomers into polymers and enhance the degree of polymerization. As the final curing time increases, double bond conversion also increases. Hence, extending the final curing time can improve the mechanical properties of 3D-printed denture-base materials. The flexural strength of 3D-printed materials increases with the final curing time [38]. This also supports our findings. In the present study, the post-curing time was 30 + 30 min, which is longer than the post-curing time in similar studies in the literature. This difference explains why the 3D-printed specimens exhibited higher flexural strength.

The superior flexural strength values of the 3D-printed resin bases amongst all the groups in this study support the notion that 3D printers could become more widely employed in future denture-base production [35].

During clinical usage, the mechanical properties of prosthetic restorations are often adversely affected by intraoral conditions involving mechanical stresses, thermal variations, and occlusal loads. The degrading effects of temperature fluctuations necessitate compensatory measures to improve the clinical lifespan of denture-base materials because of their impact on prosthetic-base materials. Routine activities like eating, drinking, and breathing result in temperature changes in the oral cavity. This can have a negative impact on the expansion of developed cracks, thus altering the physical, mechanical, and surface properties of the material over extended periods [39]. Therefore, to evaluate the clinical usability of dental materials fully, they must be tested under conditions similar to those that they face within the oral environment. Thermal cycling induces water absorption in denture-base resins, leading to degradation of polymeric chains and weakening of mechanical properties [40]. The results of this study indicate that the effect of thermal cycling varied depending on the material. Statistically, the specimens in the FL and IB groups were affected by thermal cycling, whereas those in the MD group were not affected. This difference can be attributed to differences in the chemical composition of the tested materials. Conventional heat-polymerized acrylics, produced using conventional methods, might be more resilient against temperature changes compared to milled and 3D-printed denture-base materials.

Prepolymerized PMMA is prone to water absorption because of its molecular polarity. Water absorption occurs through a diffusion mechanism among the polymer chains, and may damage the bonding within polymer networks [41]. Thermal cycling constitutes repeated sorption/desorption cycles that may result in microfractures in the PMMA matrix [42]. Although denture-base materials with low solubility are used because they release fewer monomers, unreacted monomers and water-soluble additives may leach out over long periods of thermal cycling. The heightened sensitivity of pre-polymerized PMMA to strength degradation after thermal cycling may be due to these effects [43].

We found that the flexural strength of the 3D-printed denture-base resin was also affected by thermal cycling. This change might have been caused by the material composition, inconsistencies in the printed layers, or water absorption related to heat from the manufacturing process [44].

This study had several limitations. First, variations in the printing angle could have affected the properties of the additively manufactured denture-base resin. Second, while thermal cycling was conducted using distilled water, artificial saliva might have been a more appropriate choice, as this would have been closer to clinical conditions. Different results might have been obtained with water versus saliva following long-term thermal cycling. Third, in this study, we tested only one type of product for each production technique. Further studies should include more brands representing each group. Fourth, the simple sample geometry limits the clinical usefulness of this study. Studies using specimens with more complex shapes such as those likely to be encountered in clinical situations should be conducted. Finally, 3D printers are intricate systems with many variables, and use of these printers for denture-base materials is a relatively new endeavor. Therefore, more research is needed to address the effects of various manufacturing parameters.

## Conclusions

When considering the flexural strength of denture bases produced using different methods, those created digitally (both via subtractive and additive techniques) were found to be more durable compared to conventionally manufactured bases.

Differences were found among the materials. The group with the highest flexural strength was the 3D-printing group, followed by the CAD/CAM-milling group and then the heat-polymerized-acrylic group.

Thermal cycling generally reduced flexural strength across all groups. However, statistically significant changes were observed in the 3D-printing and CAD/CAM-milled groups, while the alteration in the heat-polymerized-acrylic group was statistically insignificant.

## Data Availability

The datasets used and analysed during the current study are available from the corresponding author on reasonable request.

## References

[1] Gad MM, Fouda SM, Arrejaıe AS, Al-Thobıty AM (2019). Comparative effect of different polymerization techniques on the flexural and surface properties of acrylic denture bases. J Prosthodont.

[2] Cheng YY, Cheung WL, Chow TW (2010). Strain analysis of maxillary complete denture with three-dimensional finite element method. J Prosthet Dent.

[3] Kawaguchı T, Lassıla LV, Baba H, Tashıro S, Hamanaka I, Takahashı Y (2020). Effect of cellulose nanofiber content on flexural properties of a model, thermoplastic, injection-molded, polymethyl methacrylate denture base material. J Mech Behav Biomed Mater.

[4] Lı P, Lambart AL, Stawarczyk B, Reymus M, Spıntzyk S (2021). Postpolymerization of a 3D-printed denture base polymer: impact of post-curing methods on surface characteristics, flexural strength, and cytotoxicity. J Dent.

[5] Tasaka A, Matsunaga S, Odaka K, Ishızakı K, Ueda T, Abe S (2019). Accuracy and retention of denture base fabricated by heat curing and additive manufacturing. J Prosthodont Res.

[6] Kattadiyil MT, Jekki R, Goodacre CJ, Baba NZ (2015). Comparison of treatment outcomes in digital and conventional complete removable dental prosthesis fabrications in a predoctoral setting. J Prosthet Dent.

[7] Bidra AS, Taylor TD, Agar JR (2013). Computer-aided technology for fabricating complete dentures: systematic review of historical background, current status, and future perspectives. J Prosthet Dent.

[8] Berman B (2012). 3-D printing: the new industrial revolution. Bus Horiz.

[9] Bosch G, Ender A, Mehl A (2014). A 3-dimensional accuracy analysis of chairside CAD/CAM milling processes. J Prosthet Dent.

[10] Grande F, Tesini F, Pozzan MC (2022). Comparison of the accuracy between denture bases produced by subtractive and additive manufac- turing methods: a pilot study. Prosthesis.

[11] Beuer F, Schweiger J, Edelhoff D (2008). Digital dentistry: an overview of recent developments for CAD/CAM generated restorations. Br Dent J.

[12] Abduo J, Lyons K, Bennamoun M. Trends in computer-aided manufacturing in prosthodontics: a review of the available streams. Int J Dent. 2014;783948. 10.1155/2014/783948 PMC4000974 24817888

[13] Van Noort R (2012). The future of dental devices is digital. Dent Mater.

[14] Takamiya AS, Monteiro DR, Marra J, Compagnoni MA, Barbosa DB (2012). Complete denture wearing and fractures among edentulous patients treated in university clinics. Gerodontology.

[15] Zappini G, Kammann A, Wachter W (2003). Comparison of fracture tests of denture base materials. J Prosthet Dent.

[16] ADA. ANSI/ADA Standard No. 139 (ISO 20795-1), Denture Base Polymers. American Dental Association; 2013. Available at: https://webstore.ansi.org/Standards/ISO/ISO207952013?gclid=EAIaIQobChMI5eGNoJ_64AIVT57 ACh0YsAsyEAAYAiAAEgKeaPD_BwE.

[17] Abdulwahhab SS (2013). High-impact strength acrylic denture base material processed by autoclave. J Prosthodont Res.

[18] Gharechahi J, Asadzadeh N, Shahabian F, Gharechahi M (2014). Flexural strength of acrylic resin denture bases processed by two different methods. J Dent Res Dent Clin Dent Prospects.

[19] Hırajıma Y, Takahashı H, Mınakuchı S (2009). Influence of a denture strengthener on the deformation of a maxillary completed denture. Dent Mater.

[20] Dıaz-Arnold AM, Vargas MA, Shaull KL, Laffoon JE, Qıan F (2008). Flexural and fatigue strengths of denture base resin. J Prosthet Dent.

[21] Chhabra M, Nandıtha Kumar M, Raghavendra Swamy KN, Thıppeswamy HM (2022). Flexural strength and impact strength of heat-cured acrylic and 3d printed denture base resins- a comparative in vitro study. J Oral Biol Craniofac Res.

[22] Al-Dwaırı ZN, Tahboub KY, Baba NZ, Goodacre CJ (2020). A comparison of the flexural and impact strengths and flexural modulus of cad/cam and conventional heat-cured polymethyl methacrylate (PMMA). J Prosthodont.

[23] Lourinho C, Salgado H, Correia A, Fonseca P (2022). Mechanical properties of polymethyl methacrylate as denture base material: heat-polymerized vs. 3D-printed—systematic review and meta-analysis of in vitro studies. Biomedicines.

[24] Al-Dwaırı ZN, Tahboub KY, Baba NZ, Goodacre CJ, Özcan M (2019). A comparison of the surface properties of CAD/CAM and conventional polymethylmethacrylate (PMMA). J Prosthodont.

[25] Abualsaud R (2022). Flexural strength of CAD/CAM denture base materials: systematic review and Meta-analysis of In-vitro studies. J Int Soc Prev Community Dentistry.

[26] Alt V, Hannıg M, Wostmann B (2011). Fracture strength of temporary fixed partial dentures: CAD/CAM versus directly fabricated restorations. Dent Mater.

[27] Edelhoff D, Beuer F, Schweıger J (2012). CAD/CAM-generated high-density polymer restorations for the pretreatment of complex cases: a case report. Quintessence Int.

[28] Aguırre BC, Chen JH, Kontogıorgos ED, Murchıson DF, Nagy WW (2020). Flexural strength of denture base acrylic resins processed by conventional and CAD- CAM methods. J Prosthet Dent.

[29] Ayman AD (2017). The residual monomer content and mechanical properties of CAD \CAM resins used in the fabrication of complete dentures as compared to heat cured resins. Electron Physician.

[30] Srınıvasan M, Kalberer N, Kamnoedboon P, Mekkı M, Durual S, Özcan M (2021). CAD-CAM complete denture resins: an evaluation of biocompatibility, mechanical properties, and surface characteristics. J Dent.

[31] KattadıyıL MT, Alhelal A (2017). An update on computer-engineered complete dentures: a systematic review on clinical outcomes. J Prosthet Dent.

[32] Shım JS, Kım JE, Jeong SH, Choı YJ, Ryu JJ (2020). Printing accuracy, mechanical properties, surface characteristics, and microbial adhesion of 3D-printed resins with with various printing orientations. J Prosthet Dent.

[33] Revılla-Leon M, Özcan M (2019). Additive Manufacturing technologies used for Processing polymers: current status and potential application in Prosthetic Dentistry. J Prosthodont.

[34] Gao H, Yang Z, Lın WS, Tan J, Chen L (2021). The effect of build orientation on the dimensional accuracy of 3d-printed mandibular complete dentures manufactured with a multijet 3D printer. J Prosthodont.

[35] Prpic ´ V, Schauperl Z, ´ atic ´ C (2020). Comparison of mechanical properties of 3D-Printed, CAD/CAM, and conventional denture base materials. J Prosthodont.

[36] Andreescu C, Ghergic D, Botoaca O, Hancu V, Banateanu AM, Patroi D (2018). Evaluation of different materials used for fabrication of complete digital denture. Mater Plast.

[37] Aati S, Akram Z, Shrestha B, Patel J, Shih B, Shearston K (2022). Effect of post-curing light exposure time on the physico–mechanical properties and cytotoxicity of 3D-printed denture base material. Dent Mater.

[38] Kim D, Shim JS, Lee D, Shin SH, Nam NE, Park KH (2020). Effects of post-curing time on the mechanical and color properties of three-dimensional printed crown and bridge materials. Polym.

[39] Ayaz EA, Bağış B, Turgut S (2015). Effects of thermal cycling on surface roughness, hardness and flexural strength of polymethylmethacrylate and polyamide denture base resins. J Appl Biomater Funct Mater.

[40] Çakmak G, Donmez MB, Akay C, Abou-Ayash S, Schimmel M, Yilmaz B (2023). Effect of Thermal Cycling on the Flexural Strength and hardness of New‐Generation denture base materials. J Prosthodont.

[41] Al-Rifaiy MQ (2010). The effect of mechanical and chemical polishing techniques on the surface roughness of denture base acrylic resins. Saudi Dent J.

[42] Amin F, Iqbal S, Azizuddin S (2014). Effect of disinfectants on the colour stability of heat cure acrylic resin. J Ayub Med Coll Abbottabad.

[43] Atalay S, Çakmak G, Fonseca M, Schimmel M, Yilmaz B (2021). Effect of thermocycling on the surface properties of CAD-CAM denture base materials after different surface treatments. J Mech Behav Biomed Mater.

[44] Dimitrova M, Vlahova A, Hristov I, Kazakova R, Chuchulska B, Kazakov S (2023). Evaluation of Water Sorption and solubility of 3D-Printed, CAD/CAM milled, and PMMA denture base materials subjected to Artificial Aging. J Compos Sci.

